# Peculiarities of Aluminum Anodization in AHAs-Based Electrolytes: Case Study of the Anodization in Glycolic Acid Solution

**DOI:** 10.3390/ma14185362

**Published:** 2021-09-17

**Authors:** Lidia Zajączkowska, Małgorzata Norek

**Affiliations:** Institute of Materials Science and Engineering, Faculty of Advanced Technologies and Chemistry, Military University of Technology, Str. gen. Sylwestra Kaliskiego 2, 00-908 Warsaw, Poland; lidia.zajaczkowska@student.wat.edu.pl

**Keywords:** alpha-hydroxy acids (AHAs), aluminum, anodization, glycolic acid, citric acid, self-ordering regime

## Abstract

The anodization of aluminum (Al) in three alpha-hydroxy acids (AHAs): glycolic (GC), malic (MC), and citric (CC), was analyzed. Highly ordered pores in GC were obtained for the first time. However, the hexagonal cells were characterized by a non-uniform size distribution. Although common features of current density behavior are visible, the anodization in AHAs demonstrates some peculiarities. The electric conductivity (σ) of 0.5 M GC, MC, and CC electrolytes was in the following order: σ(CC) > σ(MC) > σ(GC), in accordance with the acid strength pK_a_(CC) < pK_a_(MC) < pK_a_(GC). However, the anodization voltage, under which a self-organized pore formation in anodic alumina (AAO) was observed (U_max_), decreased with increasing pK_a_: U_max_(CC) > U_max_(MC) ≥ U_max_(GC). This unusual behavior is most probably linked with the facility of acid ions to complex Al and the active participation of the Al complexes in the AAO formation. Depending on the AHA, its tendency and different modes to coordinate Al ions, the contribution of stable Al complexes to the AAO growth is different. It can be concluded that the structure of Al complexes, their molecular mass, and the ability to lose electrons play more important roles in the AAO formation than pK_a_ values of AHAs.

## 1. Introduction

The anodization of aluminum is one of the most studied electrochemical processes owing to its ability to produce a regular porous structure with tunable pore geometry [1,2]. The anodic aluminum oxide (AAO) resulted from the process can be applied as a membrane for chemical separation [3], in sensors [4], capacitors [5], high-density magnetic recording media [6], etc., or can serve as a template to produce other nanostructured materials with desired morphology and properties [7,8]. Generally, regular hexagonal pore arrays can be obtained under high-current-density conditions, which are mostly defined by anodization potential, temperature, and electrolyte concentration [9,10]. The application of a low electrolyte temperature and relatively high electrolyte concentration will make the high-current-density anodization proceed without burning [11]. Moreover, the hexagonal arrangement of pores is usually formed under anodizing voltage (U_max_), which is close, but not greater than the so-called critical voltage (U_c_), above which a dielectric breakdown occurs (U_max_ < U_c_) [12,13]. In other words, high electrolyte concentration, low anodizing temperature, and the applied voltage close to U_c_ (U_max_) will favor the pores to organize into the hexagonal close-packed structures (self-ordering regime). The U_max_, in turn, regulates the interpore distance (*D_c_*) in AAO. It was observed that the *D_c_* is linearly proportional to the anodizing potential, with proportionality constants of about 2.5 nm/V for mild anodization (MA) conditions [14,15]. The higher the applied voltage, the larger the *D_c_* can be obtained. On the other hand, it is quite well established that, for given anodizing conditions (concentration, temperature, etc.), the magnitude of U_max_ is determined mainly by the strength of an acid in solution [10,13,16,17]. Generally, the higher dissociation constant (pK_a_ = −log_10_K_a_) of the acid will translate into fewer acid anions in the electrolyte, and as a result, a greater U_max_ can be applied during anodization without a burning phenomenon. Therefore, the dissociation constant of acids also plays a key role in determining the interpore distances in AAO. Since relatively small variations of acid concentration have a negligible effect on anodization compared with the impact of the dissociation constant [18], the magnitude of the U_max_ usually applied during anodization in the three most studied electrolytes: 0.3 M sulfuric, 0.3 M oxalic, and 0.1–0.3 M phosphoric, increased from 25 [19] and 40 [20] to 195 V [10,21,22], respectively, owing to the following order of pK_a_: pK_a_(H_2_SO_4_) < pK_a_(H_2_C_2_O_4_) < pK_a_(H_3_PO_4_) (H_2_SO_4_ ionizes completely in aqueous solutions, pK_a_ (H_2_C_2_O_4_) = 1.3, and pK_a_ (H_3_PO_4_) = 2.1 at 25 °C [23]). From this point of view, it can be deduced that to produce AAO with a *D_c_* larger than that obtained in the H_3_PO_4_ solutions, the acids with pK_a_ > pK_a_ (H_3_PO_4_) should be selected. Hence, weak organic acids seem to be the best choice. Anodization in various organic acids, such as tartaric acid [24], squaric acid [25], or acetylenedicarboxylic acid [26], has already been studied. Among organic acids, alpha-hydroxy acids (AHAs) offer a very promising route for environmentally friendly anodization of aluminum to prepare large-interpore, regular AAO matrices.

Alpha-hydroxy acids (AHAs) are organic acids with a hydroxyl group (–OH) attached to the α carbon [27,28]. Their pK_a_ > 3 [23,29], and thus they are considered as weak acids. The AHAs are commonly used in food preservation but are also preset as natural food components [30,31]. To this family belong glycolic (GC), malic (MC), and citric (CC) acid, which contains 1, 2, and 3 carboxyl groups (–COOH), respectively, in their molecular structure. Glycolic acid is monoprotic, whereas malic and citric are diprotic and triprotic acids, which means that they can lose two and three protons in the solution, respectively. Since the second proton (positively charged) is removed from negatively charged species, the first pK_a_ (pK_a1_) is always the smallest, followed by the second, etc. Thus, the pK_a1_ can be considered as the most important chemical parameter that determines anodization conditions, mainly U_max_. However, depending on the pH of a solution and the difference between the following pK_a_ of a given acid, each species can be present to some extent in the solution [32]. Consequently, a role of the second-stage dissociation constant was also considered in the pore formation process [18,33,34].

Citric acid’s pK_a1_(C_6_H_8_O_7_) = 3.1 at 25 °C [23], and therefore comparing with H_3_PO_4_, it should be possible to apply a considerably higher value of U_max_. Formation of AAO in the citric acid solutions was studied before by several groups [35,36,37,38], but AAO with the close-packed hexagonal structure and large period (up to ca. 900 nm) was produced during anodization in high citric acid solution (1.5 M), at low temperature (0 °C) and under high anodizing voltage (400 V) [39,40]. Under this condition, the process showed some new characteristics and was called Janus anodization (JA) [39]. In this process, the current density (*i_a_*) vs. time transients demonstrated the stages typical for MA, but the passage to the following stages took a much longer time (a very slow pore nucleation). The *i_a_* was also much higher than that observed during MA, which made JA process similar to hard anodization (HA). Furthermore, during the self-ordering process, a change of the AAO color from grey to black was observed and was ascribed to a massive incorporation of citric anions into the alumina framework [39,40]. The models related to the pore growth mechanisms under the MA and HA conditions were well described in previous works [8,41]. The malic acid’s pK_a1_(C_4_H_6_O_5_) = 3.5 at 25 °C [23]. The anodization in MC was performed in various conditions [42,43,44]. The self-ordered pores in AAO were produced at 5 °C in 0.5 M malic acid solutions at 250 V after a prolonged anodization time (6 h) [44]. In both cases (citric and malic anodization), the commencement of the *i_a_* growth to a maximal value (*i_a_^max^*) was ascribed to the beginning of the pore formation process [39,43,44]. However, the *i_a_^max^* appeared after about 4 h during anodization in MC electrolyte, signifying that the nucleation in MC solution is extremely slow as compared to that conducted in CC electrolytes, where the *i_a_^max^* happened after a few minutes of anodization [39,40]. Kikuchi et al. [43] have demonstrated that the pore nucleation in malic acid initiates at grain boundaries of the aluminum. Before reaching the *i_a_^max^*, the pores formed islands separated by flat regions where no concaves on the Al surface were present. The concaves were spread over the entire Al substrate only after the *i_a_^max^* peak, which was accompanied by a slow decrease of the current [43]. Glycolic acid’s pK_a1_(C_2_H_4_O_3_) = 3.8 at 25 °C [23], and therefore, it can be anticipated that a stable, self-organized growth of AAO will be possible under a U_max_ higher than that applied during anodization in citric and malic electrolytes. The electrochemical oxidation of Al foil in glycolic acid was performed by Chu et al. [42]. The process was carried out in 1 wt% solution, at 150 V and 283 K, thus far away from the conditions where a self-ordering regime should be expected. Moreover, no detailed analysis of the process in GC was presented. The results so far obtained suggest that the anodization in AHAs electrolytes needs deeper studies, especially in terms of the significance of pK_a_ and its influence on a proper selection of anodizing parameters.

In this work, Al anodization in GC, MC, and CC electrolytes performed within self-ordering regimes is discussed and compared. The anodization in GC solution under self-organized conditions was accomplished for the first time. Highly ordered pores were formed in 0.5 M GC, at 225–250 V and 5 °C. However, the hexagonal cells on the Al substrate, obtained after the dissolution of the formed oxide, were characterized by a non-uniform size distribution. Anodization at U_max_ > 250 V was characterized by an extremely high current generated during the process and a fast consumption of Al substrate. Moreover, it was shown that the U_max_ applied during the anodization in the AHAs decreases with the increasing pK_a_ of the acids. This unusual behavior was discussed, taking into consideration the possible participation of ionic species in AAO formation and their strong ability to form stable complexes with Al.

## 2. Materials and Methods

High-purity Al foil (99.9995% Al, Goodfellow, UK) with a thickness of about 0.25 mm was cut into rectangular specimens (2 × 1 cm). Before the anodization process, the Al foils were degreased in acetone and ethanol and subsequently electropolished in a 1:4 mixture of 60% HClO_4_ and ethanol at 0 °C, under a constant voltage of 25 V, for 2.5 min. Next, the samples were rinsed with distilled water, ethanol, and dried. The as-prepared Al specimens were insulated at the back and the edges with acid-resistant tape and served as the anode. A Pt grid was used as a cathode, and the distance between both electrodes was kept constant (ca. 5 cm). The Pt/Al electrode area ratio was about 25. High purity citric acid was purchased from Chempur (Piekary Śląskie, Poland). Glycolic acid for synthesis was purchased from Sigma-Aldrich (Darmstadt, Germany). A large, 1L electrochemical cell and cooling bath thermostat (model MPC-K6, Huber company, Offenburg, Germany) were employed in the anodizing process. An adjustable DC power supply with a voltage range of 0–300 V and current range of 0–5 A, purchased from NDN, model GEN750_1500 TDK Lambda, TDK Co. Tokyo, Japan, was used to control the applied voltage. A RIGOL DM 3058E digital multimeter (Beaverton, OR, USA) was used to measure and transfer the registered current to a computer. Alumina was chemically removed using a mixture of 6 wt% phosphoric acid and 1.8 wt% chromic acid at 60 °C for 120 min.

Morphological analysis was made using a field-emission scanning electron microscope FE-SEM (AMETEK, Inc., Montvale, NJ, USA). The layer thickness of AAO was determined from three measurements taken at different areas in the secondary electrons (SE) image of a cross-sectional view of AAO. Finally, an average of the three measurements was given as a result. To obtain the interpore distance (*D_c_*) of the fabricated samples, fast Fourier transforms (FFTs) were generated based on three SEM images taken at the same magnification for every sample and were further used in calculations with WSxM software (version 5.0) [45]. The average *D_c_* was estimated as an inverse of the FFT’s radial average abscissa from three FE-SEM images for each sample.

The conductivity of the electrolytes was measured in a thermostatic cell with Elmetron CC 505 conductivity meter, Zabrze, Poland. As a result, an average value from three measurements is provided.

## 3. Results and Discussion

**Figure 1:**
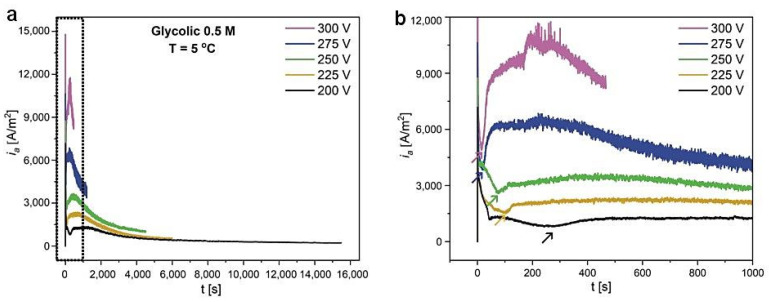
Current density (*i_a_*) versus time (*t*) transients for the samples anodized in 0.5 M glycolic acid water solution, at T = 5 °C, under different voltage (**a**); a larger magnification of the *i_a_*(*t*) curve (delineated by black, dotted rectangle) (**b**).

Anodization of aluminum was conducted in 0.5 M GC water solution at a temperature of 5 °C. In Figure 1a,b, the current density (*i_a_*) vs. time (*t*) curves are shown for different values of anodizing voltage. At U > 250 V, the extremely high *i_a_* made it impossible to perform the process for a longer time owing to a fast consumption of Al substrate. Therefore, the anodization at 300 and 275 V were stopped after 8 and 20 min, respectively. As the applied voltage decreased, the currents became less violent (*i_a_* significantly decreased), and at U = 200 V, it was possible to conduct a stable anodization for more than 4 h. The current evolution is similar to that observed during Al anodization in citric acid electrolytes [39,40]. As in CC electrolytes, the *i_a_*(*t*) curves first increase to high current peaks, followed by their decrease to a minimum, and then slowly grow to a maximal value (*i_a_^max^*). After reaching the *i_a_^max^*, the currents continuously decrease to a steady-state value. The turnover points were associated previously with various stages of pore nucleation and growth [39,40]. The minimum (marked by small arrows in Figure 1b) appeared later as the applied voltage decreased, giving an indication that the pore development was delayed owing to a smaller external electric force operating under a given electrochemical condition.

**Figure 2:**
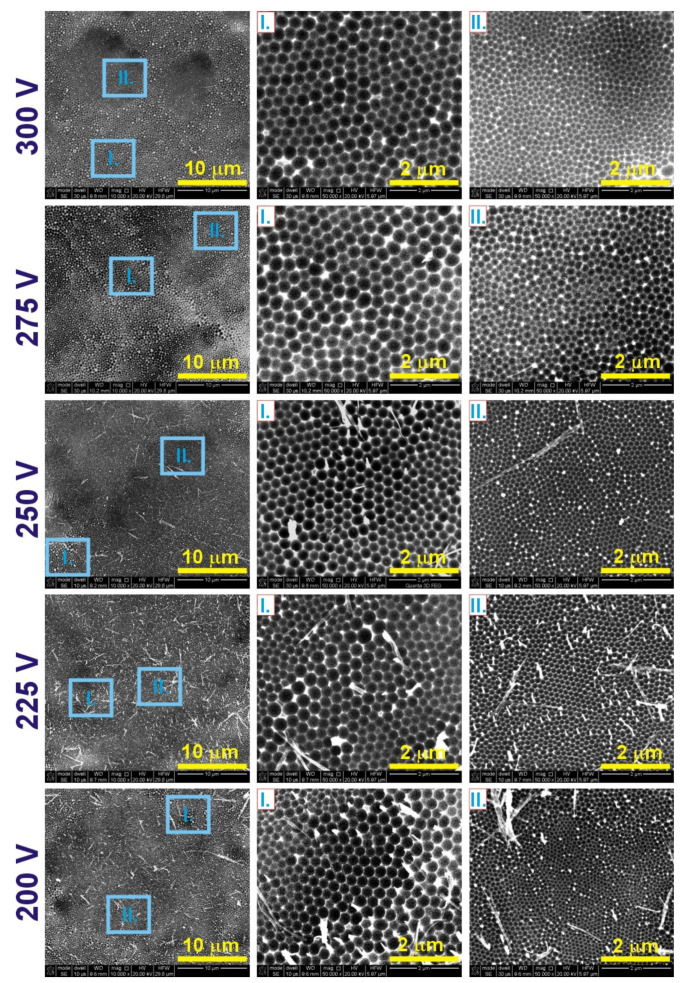
SEM images of the Al substrates after the dissolution of AAO obtained during anodization in 0.5 M glycolic acid solution at 5 °C.

The pits formed on Al substrate after the AAO dissolution, being an imprint of the pores’ bottom in AAO, are shown in Figure 2. The pores organize into a few areas containing cells of various sizes. SEM images in Figure 2 show a larger region of a given sample (first column) and a magnification of the areas with two extreme cell sizes, designated as I (second column) and II (third column). In Table 1, the interpore distance (*D_c_*) values that correspond to the center-to-center distance between neighboring cells for both I and II areas are gathered. It is observed that after anodization at 300 and 275 V (the extremely high currents), only the pores from area I exhibit the features of hexagonal ordering typical for the AAO matrix. Area II demonstrates a rather poor pore organization. At 250 V, pores in both areas are organized into a close-packed hexagonal structure. Starting from 225 V, the pore arrangement seems to be weakened in area I, whereas it is still very good in area II. The interpore distance (*D_c_*) in both areas tends to decrease with anodization voltage (Table 1). Moreover, as the applied voltage decreases, the *D_c_* in area II becomes successively closer to that obtained during anodization in 0.3 M oxalic acid solution at voltages 40–60 V [15,21].

It is worth noticing that the barrier layer is not interrupted by the extremely high currents generated during the process: hexagonally arranged corrugations are present even on Al substrates produced at 275 and 300 V. The *i_a_*(*t*) curves for all anodized samples show a similar evolution. After reaching the *i_a_^max^*, the current decreases and stabilizes at a certain value (the greater for larger applied voltage), with no characteristic sudden, rapid, and continuous rise, typical for dielectric breakdown [43,44]. This behavior was also observed by Ma et al. [39,40] during anodization in citric acid under high voltage and concentration. Instead of the dielectric breakdown, the high current density anodization was accompanied by a continuous improvement of pore arrangement and massive incorporation of citric anions (formation of black oxides).

**Figure 3:**
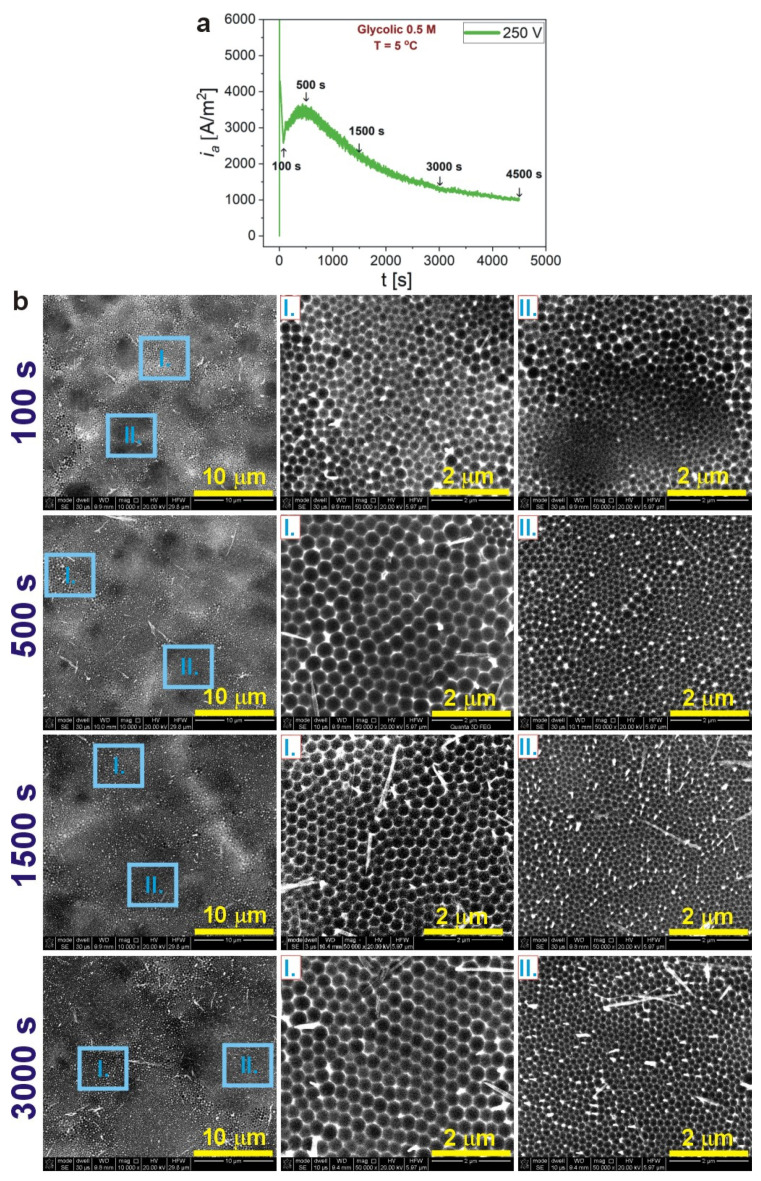
Current density (*i_a_*) versus time (*t*) transients for the samples anodized in 0.5 M glycolic acid solution, at 250 V and T = 5 °C (the arrows indicate the time after which the anodization was stopped and the sample was analyzed by SEM) (**a**); corresponding SEM images of the Al substrates after the dissolution of AAO obtained during anodization in 0.5 M glycolic acid solution at 250 V, 5 °C for different anodizing time (**b**).

Pore evolution at various stages of anodization was studied at 250 V. The morphology of the Al substrates was analyzed after 100, 500, 1500, and 3000 s of anodization (Figure 3a) and subsequent removal of AAO (the pore arrangement on the Al substrate after 4500 s of anodization are shown in Figure 2). As can be seen, in the process conducted in 0.5 M GC electrolyte, the pores are already formed after 100 s (the first minimum in the *i_a_*(*t*) curve). However, their organization is rather poor in both areas I and II. The peak that appears at 500 s of the process (*i_a_^max^*) signals the commencement of pore organization into the hexagonal arrays. As the process proceeds, the hexagonal arrangement becomes better and better. The pore evolution is generally the same as the one observed during anodization of Al in CC. Nevertheless, owing to much lower current densities reached during the process conducted in the CC electrolyte, the nanodents on aluminum were still unregularly arranged after reaching the *i_a_^max^* [39,40]. This suggests that the mechanism of the AAO formation is very similar in both electrolytes.

**Figure 4:**
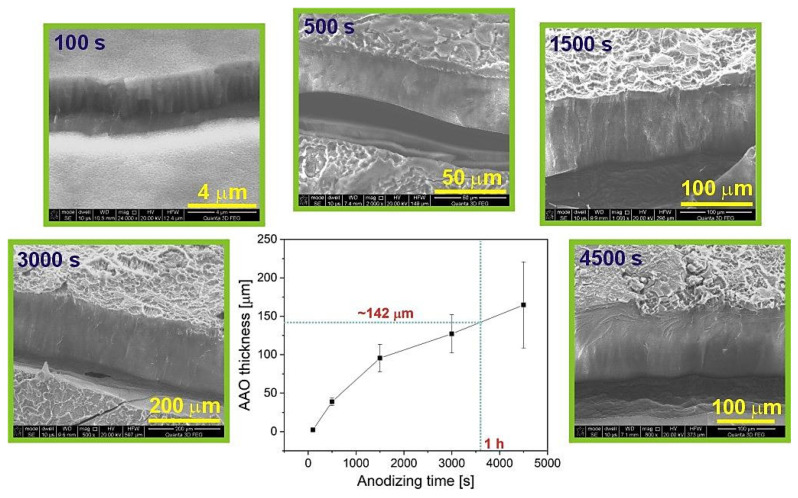
AAO thickness as a function of anodizing time; in the middle of the SEM images, the graph shows the dependence of the average AAO thickness on anodizing time.

Cross-sectional views of the AAO grown at different anodization stages are shown in Figure 4. As can be observed, the growth of AAO is very rapid, especially at the beginning of the process and slows down after approximately 2000 s. This is an effect of the extended diffusion path along the pores (diffusion-limitation) as the thickness of AAO exceeds 100 μm. The same phenomenon was observed during the hard anodization in oxalic acid [46]. The thickness of the resulted AAO is not uniform along the entire AAO cross-section, and this heterogeneity increases with anodization time (the graph in Figure 4 shows an average of three film thickness values that were measured at three different locations along a given AAO cross-section). Furthermore, it can be seen that the continuity of the top part in AAO becomes disrupted as the anodization proceeds. After 100 s the top layer is still smooth without cracks (Figure 4). However, after reaching the current peak at 500 s, when the reorganization of pores into various domains occurs, the surface of the AAO membrane begins to crush and delaminate. This effect happens because of the extremely rapid and inhomogeneous growth of AAO and huge stresses generated throughout the whole film. After 1 h of anodization, the AAO thickness is already ca. 142 μm. The thickness of self-organized AAO that was obtained in various electrolytes and electrochemical conditions are presented in Table 2. From the analysis, it appears that despite its highest pK_a1_ value (the acids in Table 2 are arranged according to the increasing pK_a1_ value) the process conducted in GC is one of the most violent. The AAO growth rate is lower even during HA in oxalic acid solution. The process is also the fastest among the ones performed in other AHAs electrolytes: the AAO thickness is about 50 μm after 1-h anodization in 1.5 M CC at 400 V, and ~165 μm after 6-h anodization in 0.5 M MC at 250 V.

**Figure 5:**
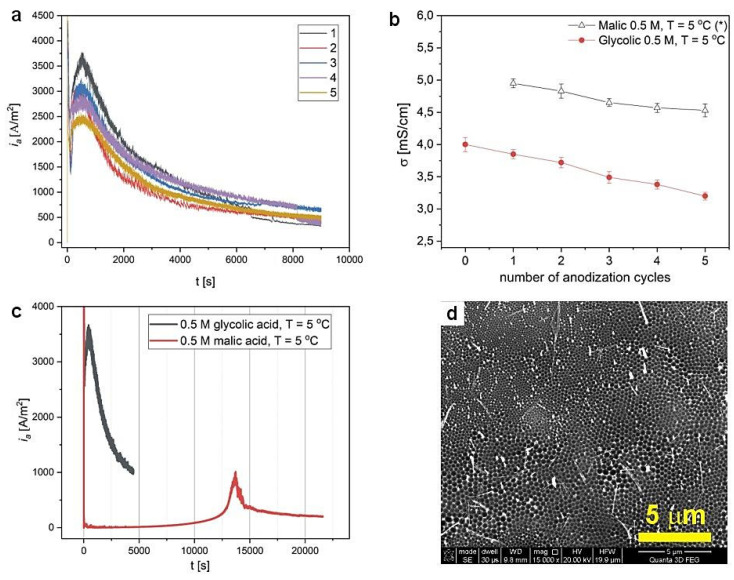
*i_a_*(*t*) curves recorded during anodization in 0.5 M glycolic acid solution, at 250 V and T = 5 °C, five times in a row (**a**); electric conductivity (σ) as a function of anodization cycles (the data for malic acid comes from ref. [44]) (**b**); a comparison of the *i_a_*(*t*) curves recorded during anodization in GC and MC electrolytes under the same conditions (the data for malic acid comes from ref. [44]) (**c**); SEM image of Al substrate after process no. 5 (**d**).

In our previous work, we have observed a very fast aging of malic acid electrolytes when the anodization was repeated in the same MC solution several times in a row, which was manifested in a visible change in current density vs. time transients and in a continuous decrease of electric conductivity (σ) [44]. A similar experiment was performed in the glycolic acid electrolyte. In Figure 5a, *i_a_*(*t*) curves were recorded during 2.5-h anodization in 0.5 M GC solution, at 250 V and T = 5 °C, five times in a row (the processes no. 1–5), are demonstrated. As can be seen, the current density drops in every subsequent cycle. Similar to the conductivity behavior in the MC electrolyte, the σ is also decreasing as the number of anodization cycles increases (Figure 5b), suggesting a decreasing amount of ionic species in the electrolyte and their transition from the solution to AAO matrix. Moreover, in accordance with the larger dissociation constant of GC over MC (pK_a1_(C_2_H_4_O_3_) = 3.8 > pK_a1_(C_4_H_6_O_5_) = 3.5), the σ(C_2_H_4_O_3_) < σ(C_4_H_6_O_5_) (anodization cycle = 0 for GC electrolyte means that the σ was measured for a freshly prepared solution). In Figure 5c, the *i_a_*(*t*) curves for the samples anodized in 0.5 M GC and MC electrolytes, at the same temperature (5 °C) and anodizing voltage (250 V), are shown. As can be seen, despite the lower σ of GC, AAO growth is much more rapid in the GC electrolyte when compared to that in the MC electrolyte: the *i_a_^max^* was reached at about 8 min in GC, whereas in MC, it appears only after ca. 4 h. In Figure 5d, an SEM image of the Al concaves after the anodization no. 5 is shown. It is visible that the concaves do not change with either a twofold increase of the anodization time or with decreasing σ in the successive cycle. As a result, the AAO possesses a complex morphology with close-packed hexagonal cells that are grouped by their different sizes into separate areas. The cells in area I are more than two times larger than the cells in area II (Table 1).

Porous membranes built of pores of various sizes are needed to study the size effect of various nanostructures on their functional properties. AAO with a gradient distribution of pore diameter was prepared by specially designed experiments involving nonparallel arrangement between an aluminum sheet and a cathode [52] or by the bipolar electrochemical anodization route [53]. In the first work, a change in interpore distance from 300 to 250 nm was obtained [52]; in the second work, a continuous change in interpore distance from ~171 to ~83 nm over a range of 5 mm on the aluminum sheet was achieved [53]. Although in the AAO produced in the GC electrolyte, there is no continuous change of *D_c_* (the areas of different pore sizes are distributed rather stochastically), it should be noted that the various cell sizes were obtained in a single anodization process, without the necessity of using special equipment.

**Figure 6:**
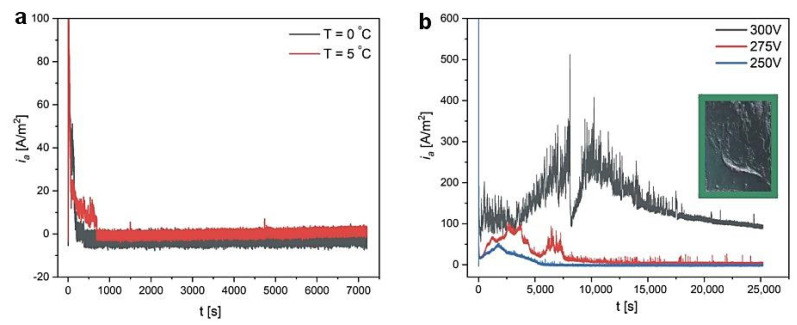
*i_a_*(*t*) curves recorded during anodization in 0.5 M citric acid water solution at 300 V and at T = 0 and 5 °C (**a**); *i_a_*(*t*) curves recorded during anodization in 1.5 M citric acid water solution at 5 °C and under various anodizing voltages (the insert shows a photograph of the sample anodized in 1.5 M CC at 300 V and 5 °C) (**b**).

Among the three AHAs: citric (CC), malic (MC), and glycolic (GC), the CC is characterized by the lowest pK_a_. According to the theory that takes the magnitude of the dissociation constant as the basic criterion for determining U_max_ (U_max_ < U_c_) [10,13,16,18,42], in this electrolyte, it should not be possible to carry out the anodization under a voltage larger than that applied during anodization in MC and GC electrolytes. Yet, a stable anodization in CC electrolyte was performed under a much higher anodizing voltage (350–400 V), and what is more, in three times larger acid concentration (1.5 M) [39,40]. In this work, the electrochemical conditions to form AAO in CC are systematically analyzed and compared directly with those applied in the GC electrolyte. In Figure 6a, *i_a_*(*t*) curves recorded during anodization in 0.5 M CC solution at 0 and 5 °C, at 300 V, are presented. As can be seen, no sign of pore formation is observed. Therefore, the acid concentration was increased to 1.5 M, resulting in a current increase after some anodizing time at 300 V, which indicated the appearance of the pore formation process. After the anodization, the oxide was dark greenish (the insert in Figure 6b) rather than black, as previously observed after anodization at 400 V and 0 °C [39,40]. In the *i_a_*(*t*) curve, two current maxima (*i_a_^max^*) of similar intensities are visible. The *i_a_^max^* is, however, much smaller than that recorded during anodization in both MC and GC electrolytes: *i_a_^max^* ~300 A/m^2^ as compared to *i_a_^max^* ~1000 A/m^2^ in MC and *i_a_^max^* ~3500 A/m^2^ in GC (see Figure 5c). When the anodizing voltage is reduced to 250 V, the *i_a_*(*t*) decreases as well (Figure 6b).

**Figure 7:**
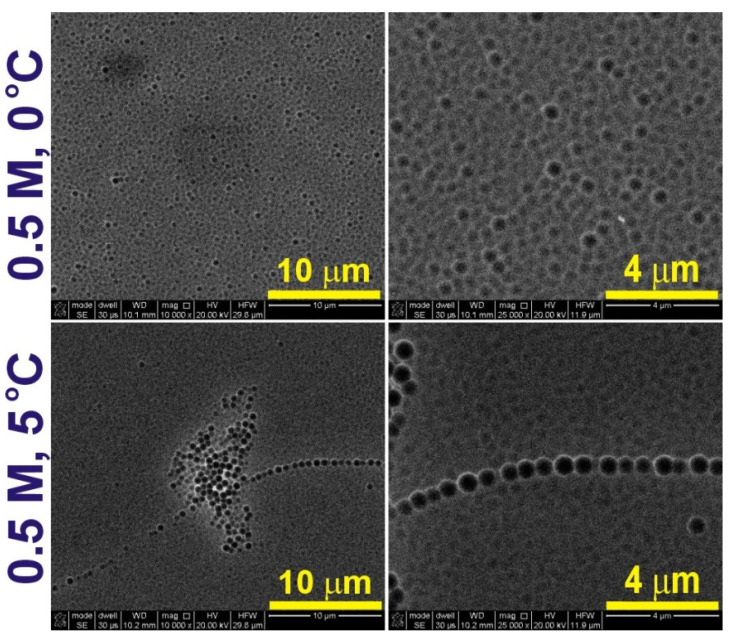
SEM images of the Al substrates after the dissolution of the oxide obtained during anodization in 0.5 M citric acid solution at 300 V and 0 and 5 °C (SEM images in the (**right**) column are larger magnifications of the respective images in the (**left**) column).

**Figure 8:**
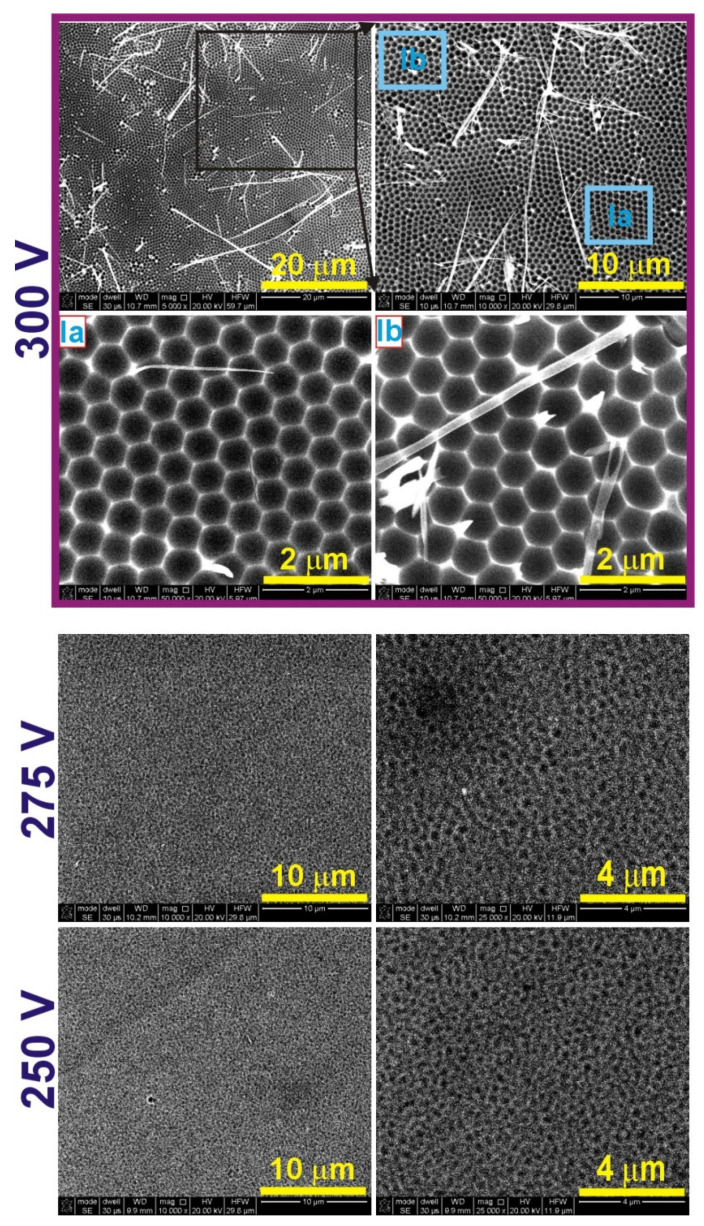
SEM images of the Al substrates after the dissolution of the oxide obtained during anodization in 1.5 M citric acid solution at 5 °C and various anodizing voltages (the SEM images in the right column of the samples anodized at 275 and 250 V are larger magnifications of the respective images in the left column).

In Figure 7, SEM images of the Al substrate obtained after oxide dissolution formed in 0.5 M CC electrolyte at the two anodizing temperatures are shown. No indentations typical for AAO originating from the pore bottom can be observed under this condition. SEM images of Al substrates after removing the oxide fabricated in 1.5 M CC, at 5 °C. and various anodizing voltages are demonstrated in Figure 8. At 300 V, the hexagonal concaves on Al are clearly visible. The pore sizes are much more uniform as compared to those produced in the GC electrolyte, although two areas can still be distinguished: one (the area Ia) with almost perfect hexagonal close-packed structure, and the second (the area Ib) with a little bit worse pore arrangement and thus a slightly larger interpore distance. In Table 3, the *D_c_* determined for both areas are presented, together with *D_c_* obtained in other AHA electrolytes at 5 °C. The *D_c_* in AAO produced in CC electrolyte is the largest compared to that reached in the other two AHA electrolytes, mostly due to the highest U_max_. However, generally, the interpore distance in AAO fabricated in AHAs is rather weakly linked with the applied voltage. Despite the same U = 250 V (and all other anodizing parameters) used in GC and MC electrolytes, the *D_c_* in AAO grown in MC is much larger than that produced in the GC electrolyte (even if one takes into account the *D_c_* values from area I). This effect is most probably associated with the complex architecture of the pores formed in GC solution (a few areas of different cell sizes), which eludes the simple proportionality between *D_c_* and U in this electrolyte. On the Al substrate fabricated at lower voltages (275 and 250 V), tiny dimples with no arrangement are visible after the oxide dissolution. This suggests that the conditions were already not sufficient to induce AAO growth with the typical long, hexagonally organized channels.

In Table 4, electric conductivity determined for the citric acid electrolytes is gathered. According to Pashchanka et al. [54], the electric conductivity is one of the crucial parameters of electrolyte compositions, with its minimum limit requirement for the self-assembly being around 4–5 mS/cm. This requirement is fulfilled for all AHAs studied in this work. The σ values of the CC electrolyte are the highest among the other AHAs (Figure 5b), as expected based on its lowest pK_a_. Furthermore, the CC electrolyte seems also to be not so prone to aging when the anodization is repeated in the same solution: the values remain stable after the subsequent anodizing process conducted in 1.5 M CC solution, at 5 °C, and under decreasing anodizing voltage (Table 4).

In Table 5, the molecular structure, pK_a_ values, electric conductivity, electrolyte concentration, and U_max_ were determined for anodization conducted at 5 °C, where the best pore ordering (close-packed hexagonal structure) was observed, are gathered. As can be seen, the conductivity decreases with the strength of the acid: for pK_a_(CC) < pK_a_(MC) < pK_a_(GC), the σ(CC) > σ(MC) > σ(GC). As was already mentioned, it is frequently assumed that the pK_a_ is the most important parameter that governs anodization (a smaller pK_a_ corresponds to more acid anions, which can obtain a higher incorporation current (*j_c_*) and thus a smaller U_max_ [10,13,16,18,42]) and even a small variation in electrolyte concentration and application of various anodizing temperature do not undermine the leading role of the pK_a_. However, this assumption seems to not hold for AHAs. Instead of the expected increase of U_max_ with decreasing the acid strength: U_max_ (CC) < U_max_ (MC) < U_max_ (GC), the U_max_ applied during anodization in the AHAs’ electrolytes appears in the following order: U_max_(CC) > U_max_(MC) ≥ U_max_(GC) (Table 5). U = 250 V was the maximum anodizing voltage to obtain regular hexagonal pore arrays in the MC electrolyte. Lowering U resulted in worsening of the pore ordering in AAO in MC [45]. In GC instead, a stable self-organized anodization was performed at U ≤ 250 V. In the CC electrolyte, a 0.5 M concentration was not sufficient for pore nucleation to begin even at 300 V, and a substantially greater concentration had to be used to induce pore self-organization. In Table S1 (Supplementary Materials), the relationship between pK_a_ and U_max_ is analyzed in more detail. Excluding the examples where no self-ordering regimes were found (anodization in acethylenedicarboxylic and squaric acid solutions), the U_max_ increases with pK_a_ of the acid with some exceptions (Table S1). Anodization in phosphonic, etidronic, and phosphonoacetic acid solutions (highlighted in grey in Table S1) actually started at a much lower voltage, which was next increased to the targeted values listed in Table S1. The anodization was then conducted under the target voltages for a predetermined time. The method, although not named hard anodization, was in fact very similar to the approach applied in the HA [46], and therefore, cannot be directly compared with the other processes gathered in Table S1. The lower than expected U_max_ in the case of malonic and tartaric electrolytes can be explained by the one order of magnitude larger acid concentration (5 M and 3 M, respectively) used in this process, which are considerably larger than that commonly used. The large concentration leads to more acid anions and higher ionic currents that induce a lower U_max_ [16,18]. Therefore, the malonic and tartaric acid anodization seems to confirm the rule: the stronger the acid, the lower the U_max_. However, the selenic acid solutions and particularly the AHA-based electrolytes are out of this trend [40,44,55,56]. These observations give an indication that other parameters (including the molecular mass of the acid species) and phenomena should be taken into consideration when aluminum is electrochemically oxidized in acidic solutions.

According to traditional theories of dielectric breakdown, the following relationship holds for the U_max_ and the breakdown voltage (U_c_) [17,18]:

(1) $$U_{\max}<U_{c}=\left(\frac{E}{\alpha }\right)\ln\left(\frac{z}{\gamma \eta }\right)=\left(\frac{E}{\alpha }\right)\ln\left(\frac{zj_{o}}{\eta j_{c}}\right)$$

where *E* is the electric field across the barrier layer that is often constant, ∼1.0 V/nm; α is the impact ionization coefficient, which is in a reciprocal relationship with the mean free path (λ) of an electron passing a distance of 1 cm (α = 1/λ), and the coefficient α will increase with the *E*; *j_o_* is the oxidation current that corresponds to inward migration of O^2^^−^ and leads to the formation of the oxide, *j_c_* is the incorporation current that comes from acid anions (*j_c_* is considered to be a constant fraction of *j_o_*, i.e., *j_c_* = *γj_o_*).

During the anodization, electrolyte species (e.g., acid anions) will migrate to the AAO barrier layer, and as a consequence of the high *E*, some of them will release primary electrons into the oxide conduction band (electronic current *j_e0_* = *ηj_c_* = *ηγj_o_*, where *γ* is determined by the concentration of electrolyte species and *η* denote the ability of the electrolyte species for releasing electron). These electrons are accelerated by the high *E* producing the avalanche electronic current (*j_e_*), which should be a fraction (*z)* of the oxidation current *j_o_*, i.e., *j_e_* = *zj_o_* = *zj_c_/γ*, with *z* ≤ 1/3 [17].

It is believed that the contribution of acid anions to the formation of AAO is negligible because the anions migrate slower than oxygen species, and thus, they require a stronger electric field to reach the oxide/metal interface in a reasonable time [57,58]. However, glycolate, malate, and citric anions seem to actively participate in the AAO growth. This was already noticed by Ma et al. [39] on the occasion of anodization in a CC solution. Based on the results, the authors concluded that the amount of free citric acid anions (i.e., H_2_Cit^−^, HCit^2−^, Cit^3−^) actually plays a crucial role in pore nucleation. Our experiments partially confirmed this conclusion: the close-packed hexagonal structure was obtained only in the higher citric acid concentration (1.5 M), whereas the pores were not formed during anodization in 0.5 M CC. The heavier ionic species require a stronger *E* to reach the reaction spot, which naturally increases U_c_ (as well as U_max_). Most probably, the relatively large molecular mass of selenic acid was responsible for the relation: U_max_(selenic) ≥ U_max_(oxalic) despite that pK_a_(selenic) < pK_a_(oxalic) (see Table S1, [55,56]). Owing to the lowest molecular mass of GC, the glycolate anions migrate easier to the barrier layer than malate or citrate anions, giving rise to a larger *z* value, which is manifested in much more intensive currents observed during anodization in GC as compared to the currents recorded during the process performed in the other two AHAs (Figure 5c and Figure 6b). Citric anions, on the other hand, as the heaviest species, require a larger anodizing voltage (300 V) to start the pore formation. Apparently, citric ions also have a lower ability to release electrons (*η*) compared to malic and glycolic species. Thus, pores are formed under larger anodizing voltages and in higher acid concentrations. In glycolic acid, on the contrary, owing to its relatively large *η*, three times lower acid concentration (*γ*) is sufficient to produce regular pore arrays under a much lower (250 V) anodizing voltage. The physical parameters of AHA electrolytes are, however, not sufficient to explain all similarities and differences of anodization in AHAs solution. Since the experimental data strongly suggest that the GC, MC, and CC anions take an active part in AAO growth, their structural features should also be taken into account. It has to be remembered that AHAs can form various complexes with Al ions. Ma et al. [39] have postulated that at the beginning of the process, a certain amount of Al^3+^ are consumed by the citrate anions to form the Al-citrate complexes, causing the *i_a_* to decrease. As the process proceeds, these complexes transform slowly to citric-acid-incorporated alumina, which randomly precipitates on barrier-type alumina to form protuberances. The high electric field concentrates between those protuberances giving rise to field-assisted oxide dissolution accompanied by the *i_a_* increase, and finally to the pore development [40]. Similar processes may occur during anodization in GC and MC electrolytes. Different molecular structures and chemical properties of citric, malate, and glycolate complexes may, in turn, modify to a different extent the field-assisted oxide dissolution process leading to the peculiar behavior of current flow during anodization, which is further reflected in the resulted AAO morphology. It was observed, for instance, that the glycolate ligand coordinates via both the carboxylate and the hydroxy group to Al^III^ ion, forming a binuclear A^lII^-glycolate complex with three hydrogen bonds connecting two *fac*-A^lII^-glycolate complexes [59]. What is more, it was shown that the Al^III^ facilitates the ionization of the hydroxy group of the glycolate. Therefore, it would not be surprising if these complexing properties of GC ions contributed to the AAO growth process giving rise to the observed peculiarities, such as extremely high currents generated during the anodization in this acid and the non-uniform cell size distribution in AAO.

## 4. Conclusions

The anodization in glycolic acid was performed under the electrochemical conditions close to the ones used during the anodization in citric and malic acid solutions, where the self-ordering regimes were operative. Anodization of Al in the three AHA electrolytes was compared. In GC, the pores organize into the hexagonal close-packed structures under the following conditions: 0.5 M, 225–250 V, 5 °C. However, they are grouped into a few areas of different cell sizes. In general, the growth of AAO in the three AHAs follows the Janus type anodization, which is characterized by the same *i_a_*(*t*) stages as in MA, but the magnitude of the generated currents is typical for HA. The peak (*i_a_^max^*) in the *i_a_*(*t*) curves, which was previously associated with the initiation of pore development, appears after a significantly different anodizing time depending on the AHA used. The process is the fastest in the GC electrolyte. The electric conductivity (σ) of 0.5 M GC, MC, and CC electrolytes decreases in accordance with the acid strength pK_a_(CC) < pK_a_(MC) < pK_a_(GC): σ(CC) > σ(MC) > σ(GC). However, the anodization voltage, under which a self-organized pore formation AAO was observed (U_max_), decreased with increasing pK_a_: U_max_(CC) > U_max_(MC) ≥ U_max_(GC). Moreover, to initiate the pore formation in CC, a three times larger concentration is required than in GC or MC electrolytes, and the *i_a_^max^* (CC) < *i_a_^max^* (MC) < *i_a_^max^* (GC). This peculiar behavior is most probably linked with the diverse propensity of acid ions to complex Al. Depending on the AHA, its tendency and ways to coordinate Al ions, the contribution of stable Al complexes to the AAO growth varies. The molecular structure of the organic ions, as well as the structure of Al complexes, their molecular mass, and ability to lose electrons, play a crucial role in the AAO formation in AHAs electrolytes and seem to be more important than the pK_a_ values of AHAs. The anodization in AHA electrolytes seems to be a promising, environmentally friendly technique to produce robust AAO films with desired anti-corrosive properties.

## Figures and Tables

**Table 1:** Interpore distance (*D_c_*) in AAO produced in 0.5 Glycolic acid solution at 5 °C and under different anodizing voltages.

	300 V	275 V	250 V	225 V	200 V
***D_c_* (nm)**	I. 397 ± 39 II. 193 ± 41	I. 408 ± 38 II. 202 ± 30	I. 369 ± 22 II. 136 ± 26	I. 305 ± 49 II. 145 ± 18	I. 295 ± 29 II. 116 ± 18

**Table 2:** Thickness of AAO obtained in various anodizing conditions (the acids are arranged according to their increasing pK_a1_).

Electrolyte, Type of the Process	Anodizing Voltage (V)	Anodizing Temperature (°C)	Anodizing Time (h)	AAO Thickness (μm)	Refs
0.3 M sulfuric acid, MA	25/25/25	1/0/1	10/4/1	~40/~15/~10	[47]/[48]/[49]
0.3 M oxalic acid, MA	40/40/40	1/0/17	16/4/1	~30/~10/~15	[47]/[48]/[49]
0.3 M oxalic acid:ethanol = 1:1, *v*/*v*, MA	40	0	24	~12	[50]
0.3 M oxalic acid, HA	140	1	1	~80	[46]
2 wt% phosphoric acid, MA	175	0	30	~55	[47]
1 wt% phosphoric acid, MA	195	0	1	~2.5	[51]
0.1 M phosphoric acid, MA	195	12	1	~6	[49]
1.5 M citric acid, JA	400	0	1	~50	[39]
0.5 M malic acid, JA	250	5	6	~162	[44]
0.5 M glycolic acid, JA	250	5	1	~142	This work

**Table 3:** Interpore distance (*D_c_*) in AAO produced in various AHAs electrolytes at 5 °C.

	Glycolic 0.5 M 250 V	Malic 0.5 M 250 V	Citric 1.5 M 300 V
***D_c_* (nm)**	I. 369 ± 22 II. 136 ± 26	527 ± 12 (*)	Ia. 605 ± 20 Ib. 677 ± 55

* from ref. [44].

**Table 4:** Electric conductivity (σ) of citric acid water solutions measured directly after the electrolyte preparation (“fresh” solution) and after the subsequent anodizing processes (arranged sequentially from up to down the Table).

			σ (mS/cm)
0.5 M	0 °C	fresh	7.42 ± 0.10
		300 V	7.39 ± 0.09
	5 °C	300 V	7.24 ± 0.07
1.5 M	5 °C	fresh	8.09 ± 0.08
		300 V	8.15 ± 0.08
		275 V	8.12 ± 0.06
		250 V	8.13 ± 0.11

**Table 5:** Molecular mass and structure, dissociation constants pK_a_ (acidity at T~25 °C) [23,29], and conductivity of α-hydroxy acids together with the concentration and U_max_ applied during anodization of Al.

Name (Molecular Mass (g/mol))	Molecular Structure	pK_a_ T~25 °C	Conductivity (mS/cm)	Concentration (M)	U_max_ (V)
Citric acid (192.12)	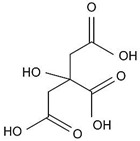	3.1 (COOH) 4.8 (COOH) 6.4 (COOH)	8.15 ± 0.08	1.5	400 V * 300 V
Malic acid (134.08)	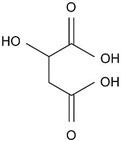	3.5 (COOH) 5.1 (COOH)	4.95 ± 0.07 **	0.5 **	250 V **
Glycolic acid (76.05)	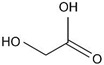	3.8 (COOH)	3.85 ± 0.07	0.5	250–225 V

* from ref. [40]; ** from ref. [44]

## Data Availability

Not applicable.

## Supplementary Materials

The following are available online at https://www.mdpi.com/article/10.3390/ma14185362/s1, Table S1: Dissociation constants (pK_a1_ and pK_a2_) at ~25 °C and molecular mass of selected acids used to produce anodic alumina (AAO) in a given electrolyte concentration, anodizing voltage, and temperature.
