# Supplementary material for: Peculiarities of Aluminum Anodization in AHAs-Based Electrolytes: Case Study of the Anodization in Glycolic Acid Solution
Source: Materials (Basel). 2021 Sep 17;14(18):5362. doi: 10.3390/ma14185362 (PMC8468596; doi:10.3390/ma14185362)
Supplement: Supplementary file 1 [file materials-14-05362-s001.zip › materials-1356416-supplementary.pdf]

materials

Supplementary Materials

Peculiarities of Aluminum Anodization in AHAs – based electrolytes: Case Study of the Anodization in Glycolic Acid Solution

Lidia Zajączkowska and Małgorzata Norek *

Institute of Materials Science and Engineering, Faculty of Advanced Technologies and Chemistry, Military University of Technology, Str. Gen. Sylwestra Kaliskiego 2, 00-908 Warsaw, Poland; lidia.zajaczkowska@student.wat.edu.pl; malgorzata.norek@wat.edu.pl
* Correspondence: malgorzata.norek@wat.edu.pl

Received: date; Accepted: date; Published: date

Table S1 Dissociation constants (pK_{a1} and pK_{a2}) at ~25 °C (unless stated otherwise) and molecular mass of selected acids used to produce anodic alumina (AAO) in a given electrolyte concentration, anodizing voltage and temperature.

Acid	pKa1<br>pKa2	Molecular Mass [g/mol]	Anodizing voltage [V]	Electrolyte concentration [M]	Anodizing temperature [°C]	Self-ordering	Refs
Sulfuric	strong [a]<br>2.0 [a]	98.08	25-30<br>25	0.3<br>0.3	-1<br>10	Yes<br>Yes	[1]<br>[2]
Selenic	strong [b]<br>1.7 [b]	144.97	48<br>42-46	0.3<br>3.0	~ 20<br>~ 0	Yes<br>Yes	[3]<br>[4]
Acethylene di-carboxylic	0.7 [c]<br>2.3 [c]	114.06	87-95	0.3	~0-60	No	[5]
Oxalic	1.3 [a]<br>4.3 [a]	90.03	40<br>40-50	0.3<br>0.3	1-5<br>35-50	Yes<br>Yes	[6]<br>[7]
Phosphonic	~1.3 [b*]<br>6.70 [b*]	80.99	150-180**	0.5-2.0	~0-10	Yes	[8]
Etidronic	1.35 [d]<br>2.87 [d]	206.03	210-270**<br>210-260**	0.3<br>0.3	~20-40<br>21-38	Yes<br>Yes	[9]<br>[10]
Squaric	1.5 [e]<br>3.5 [e]	114.06	100-120	0.1	~ 20	No	[11]
Phosphono-acetic	~2.0 [f]<br>5.11 [f]	140.03	205-225**	0.3-0.9	~10-15	Yes	[12]
Phosphoric	2.1 [a]<br>7.2 [a]	97.99	195	0.2-0.3	0-5	Yes	[6]
Malonic	2.8 [a]<br>5.7 [a]	104.06	120	5.0	5	Yes	[13]
Tartaric	3.0 [a]<br>4.4 [a]	150.09	195	3.0	5	Yes	[13]
Citric	3.1 [a]<br>4.8 [a]	192.12	300<br>300<br>400	0.5<br>1.5<br>1.5	5<br>5<br>0	No<br>Yes<br>Yes	this work<br>[14]
Malic	3.5 [a]<br>5.1 [a]	134.08	250	0.5	5	Yes	[15]
Glycolic	3.8 [a]	76.05	225-250	0.5	5	Yes	this work

* Measured at 20 °C.

** The provided values are target voltages that were reached after a linear increase of anodizing voltage for first 2.5 min of anodization and then were held constant for predetermined time (in ref. [10] the starting voltage was 80 V, which was next raised to the target values for 2-3 min).

[a] Martell, A.E.; Smith, R.M. Critical Stability Constants; Plenum Press: New York, NY, USA, 1976; Volume 1–4.

[b] Chemistry LibreTexts™. Available online: https://chem.libretexts.org/Ancillary_Materials/Reference/Reference_Tables/Equilibrium_Constants/E1%3A_Acid_Dissociation_Constants_at_25C (01.09.2021).

[c] Schwartz L. M.; Gelb, R. I.; Laufer, D. A. Aqueous dissociation of acethylenedicarboxylic acid. *J. Chem. Eng. Data* **1980**, *25*, 95-96.

[d] Glynn, G. R.; Born, J. L. An updated pKa listing of medicinal compounds. *Drug Intell. Clin. Pharm.* **1986**, *20*, 683-686.

[e] Ionization Constants of Heteroatom Organic Acids. Available online: https://www2.chemistry.msu.edu/faculty/reusch/virttxtjml/acidity2.htm (01.09.2021).

[f] Heubel, P.-H. C.; Popov, A. I. Acid properties of some phosphonocarboxylic acids. *J. Solution Chem.* **1979**, *8*, 615-625.

References

1. Fabrication of high quality anodic aluminum oxide (AAO) on low purity aluminum—A comparative study with the AAO produced on high purity aluminum. *Electrochimica Acta* **2013**, *105*, 424-432.
2. Li, A. P.; Müller, F.; Birner, A.; Nielsch, K.; Gösele, U. Hexagonal pore arrays with a 50-420 interpore distance formed by self-organization in anodic alumina. *J. Appl Phys.* **1998**, *84*, 6023-6026.
3. Nishinaga, O.; Kikuchi, T.; Natsui, S.; Suzuki, R.O. Rapid fabrication of self-ordered porous alumina with 10-/sub-10-nm-scale nanostructures by selenic acid anodizing. *Scientific reports* **2013,** *3*, 2748 (6pp).
4. Kikuchi, T.; Nishinaga, O.; Natsui, S.; Suzuki, R. O. Self-ordering behavior of anodic porous alumina via selenic acid anodizing. *Electrochimica Acta* **2014**, *137*, 728-735.
5. Kikuchi, T.; Nishinaga, O.; Natsiu, S.; Suzuki R. O. Fabrication of anodic nanoporous alumina via acetylenedicarboxylic acid anodizing, *ECS Electrochem. Lett.* **2014**, *3*, C25- C28.
6. Sulka, G.D. Highly ordered anodic porous alumina formation by self-organized anodizing. In: *Nanostructured Materials in Electrochemistry*, 1st ed.; Eftekhari A., Ed.; Wiley-VCH, Weinheim, 2008, pp. 1-116.
7. Stępniowski W. J.; Bojar, Z. Synthesis of anodic aluminum oxide (AAO) at relatively high temperatures. Study of the influence of anodization conditions on the alumina structural features *Surface and Coatings Technology* **2011**, *206*, 265-272.
8. Akiya, S.; Kikuchi, T.; Natsui, S.; Sakaguchi, N.; Suzuki, R.O. Self-ordered porous alumina fabricated via phosphonic acid anodizing, *Electrochimica Acta* **2016**, *190*, 471–479.
9. Kikuchi, T.; Nishinaga, O.; Natsui, S.; Suzuki, R.O. Fabrication of self-ordered porous alumina via etidronic acid anodizing and structural color generation from submicrometer-scale dimple array, *Electrochimica Acta* **2015**, *156*, 235–243.
10. Norek, M.; Łażewski, M. Manufacturing of highly ordered porous anodic aluminawith conical pore shape and tunable interpore distance in the range of 550 nm to 650 nm. *Mater. Sci. –Poland* **2017**, *35*, 511-518.
11. Kikuchi, T.; Yamamoto, T.; Natsui, S.; Suzuki, R. O. Fabrication of anodic porous alumina by squaric acid anodizing, *Electrochimica Acta* **2014**, *123*, 14-22.
12. Takenaga, A.; Kikuchi, T.; Natsui, S.; Suzuki, R.O. Self-ordered aluminum anodizing in phosphonoacetic acid and its structural coloration, *ECS Solid State Lett.* **20**15, *4*, P55–P58.
13. Ono, S.; Saito, M.; Asoh, H. Self-ordering of porous alumina formed in organic acid electrolytes. *Electrochim. Acta* **2005**, *51*, 827–833.
14. Ma, Y.; Wen, Y.; Li, J.; Lu, J.; Li, Y.; Yang, Y.; Feng, C.; Hao, C.; Zhang, Z.; Hu, J.; Sun, R. Pore nucleation mechanism of self-ordered alumina with large period in stable anodization in citric acid. *J. Electrochem. Soc.* **2018**, *165*, E311-E317.
15. Zajączkowska, L.; Siemiaszko, D.; Norek, M. Towards self-organized anodization of aluminum in malic acid solutions – new aspects of anodization n the organic acid. *Materials* **2020**, *13*, 3899 (15 pp).
